# Recent Advances in Nanoencapsulation Systems Using PLGA of Bioactive Phenolics for Protection against Chronic Diseases

**DOI:** 10.3390/ijerph16244962

**Published:** 2019-12-06

**Authors:** Rohanizah Abdul Rahim, Putri Ayu Jayusman, Norliza Muhammad, Fairus Ahmad, Norfilza Mokhtar, Isa Naina Mohamed, Norazlina Mohamed, Ahmad Nazrun Shuid

**Affiliations:** 1 Pharmacology Department, Faculty of Medicine, Universiti Kebangsaan Malaysia, Cheras, 56000 Kuala Lumpur, Malaysia; rohanizah@usm.my (R.A.R.); putri.ayujay@gmail.com (P.A.J.); norliza_ssp@ppukm.ukm.edu.my (N.M.); isanaina@ppukm.ukm.edu.my (I.N.M.);; 2 Advanced Medical and Dental Institute, Universiti Sains Malaysia, Bertam, 13200 Kepala Batas, Pulau Pinang, Malaysia; 3 Anatomy Department, Faculty of Medicine, Universiti Kebangsaan Malaysia, Cheras, 56000 Kuala Lumpur, Malaysia; apai.kie@gmail.com; 4 Physiology Department, Faculty of Medicine, Universiti Kebangsaan Malaysia, Cheras, 56000 Kuala Lumpur, Malaysia; norfilza@ppukm.ukm.edu.my

**Keywords:** nanoparticles, encapsulation, polyphenols, poly (lactic-co-glycolic acid)

## Abstract

Plant-derived polyphenolic compounds have gained widespread recognition as remarkable nutraceuticals for the prevention and treatment of various disorders, such as cardiovascular, neurodegenerative, diabetes, osteoporosis, and neoplastic diseases. Evidence from the epidemiological studies has suggested the association between long-term consumption of diets rich in polyphenols and protection against chronic diseases. Nevertheless, the applications of these phytochemicals are limited due to its low solubility, low bioavailability, instability, and degradability by in vivo and in vitro conditions. Therefore, in recent years, newer approaches have been attempted to solve the restrictions related to their delivery system. Nanoencapsulation of phenolic compounds with biopolymeric nanoparticles could be a promising strategy for protection and effective delivery of phenolics. Poly(lactic-co-glycolic acid) (PLGA) is one of the most successfully developed biodegradable polymers that has attracted considerable attention due to its attractive properties. In this review, our main goal is to cover the relevant recent studies that explore the pharmaceutical significance and therapeutic superiority of the advance delivery systems of phenolic compounds using PLGA-based nanoparticles. A summary of the recent studies implementing encapsulation techniques applied to polyphenolic compounds from plants confirmed that nanoencapsulation with PLGA nanoparticles is a promising approach to potentialize their therapeutic activity.

## 1. Introduction

Nowadays, the world appears to be increasingly interested in health benefits of foodstuff and its health-enhancing ingredients [1]. Bioactive compounds, which have been extensively studied for their roles in disease prevention, are extra-nutritional constituents that typically occur in small quantities in plants and food products [2]. Epidemiological studies and associated meta-analyses have strongly suggested that long-term consumption of diets rich in plant polyphenols provide protection against the development of various diseases, including cancers, cardiovascular diseases, diabetes, osteoporosis, and neurodegenerative diseases [3].

Polyphenols are secondary metabolites present in all vascular plants that have been implicated as the active components in a number of herbal and traditional medicines [4]. More than 8000 polyphenolic compounds have been identified in various plant species and several of them are known to possess a wide spectrum of pharmacological properties [5]. Fruits like grapes, apple, pear, cherries, and berries contains up to 200 to 300 mg polyphenols per 100 grams of their fresh weight. Since polyphenols can be found in a diverse range of foods (fruits, vegetables, cereals) and beverages, it has been estimated that in a normal diet, the daily intake of phenols could range from about 20 mg to 1 g, which may be as higher as the recommended intake of vitamin E [6]. Estimated dietary intakes may differ for each class of polyphenols but the total intake of polyphenols in people who eat several servings of fruits and vegetables per day probably may reach 1 g/d [7].

Most of the etiology and progression of acute and chronic clinical disorders were induced by oxidative and nitrosative stresses, which may suggest that antioxidants can display health benefits as prophylactic agents [8]. Many of the biological functions of polyphenols have been attributed to their free radical scavenging and antioxidant activities. Polyphenols exhibit a wide range of pharmacological properties, including antibacterial, anti-inflammatory, antiallergic, hepatoprotective, antithrombotic, antiviral, anticarcinogenic, and vasodilatory actions [9]. Molecular studies have revealed that polyphenols can exert modulatory actions in biological cells by the interaction with molecular targets central to the cell signaling machinery [8]. It is well established that polyphenol-rich diets may increase plasma antioxidant capacity. Polyphenols may protect cell constituents against the harmful oxidative damage and therefore, limit the risk of various degenerative diseases associated with oxidative stress.

However, the health benefit of polyphenols is not only dependent on their intake but also their bioavailability. Since metabolites reaching the blood and tissues are different from those present in food, identification of metabolites and evaluation of their biological activity is a big challenge [10]. Several studies have shown that polyphenols were extensively metabolized during transportation across the small intestine and liver, resulting in significant alteration of their redox potential [8]. Only a small proportion of the molecules remain available following oral administration, which could limit the activity and potential health benefits of polyphenols [11]. 

One of the approaches to improve the bioavailability of polyphenols is by incorporating them into nanoparticles. In recent years, nanotechnology has been rapidly expanding in the food and pharmaceutical industries, especially with the application of nanoencapsulation of bioactive compounds for biological purposes [12,13]. It involves the production, processing, and application of materials with sizes less than 1000 nm [14]. Polymeric nanoparticle is one of the most effective ways to circumvent the delivery problems of phenolics, and for protection of the nutrients against undesirable circumstances. 

Poly(lactic-co-glycolic acid) (PLGA) is a biodegradable polymer that has been successfully developed in the field of nanomedicine. It undergoes hydrolysis in the body to produce the biodegradable metabolite monomers, lactic acid and glycolic acid. These two monomers are endogenous and easily metabolized by the body via the Kreb cycle, hence there is very minimal systematic toxicity associated with the use of PLGA for biomaterial applications [15]. PLGA is approved by the US Food and Drug Administration for therapeutic use in humans and is commercially available in different molecular weights and copolymer compositions. The various forms of PLGA are identified by the monomers’ ratios. It is well suited for sustained intracellular delivery of drugs and biological macromolecules [16]. The current review summarizes relevant studies, which have explored the therapeutic feasibility and pharmaceutical significance of PLGA nanopolymers as advanced delivery systems for phenolic compounds. 

## 2. Phenolics Phytochemicals 

**Figure 1:**
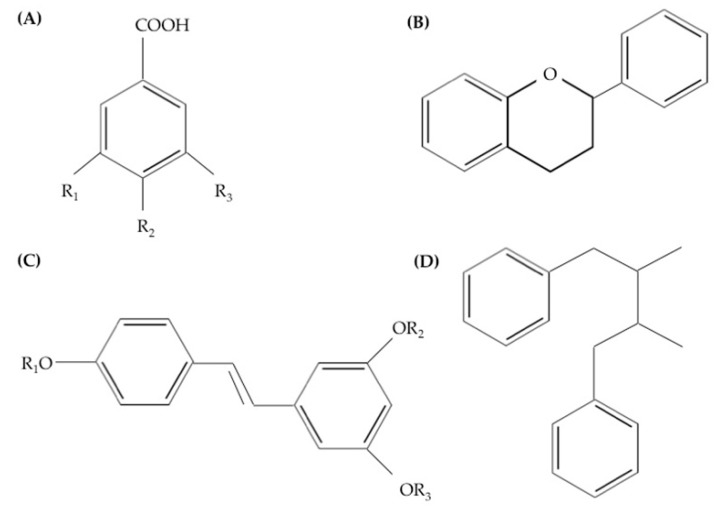
Groups of polyphenols and their basic chemical structures. (**A**) Phenolic acids, (**B**) flavonoids, (**C**) stilbenes, and (**D**) lignans.

Polyphenols may be classified into different groups based on the number of phenol rings they possess and on the basic structure that bind these rings to one another [17]. They are mainly classified as: phenolic acids (hydroxybenzoic acids and hydroxycinnamic acids), flavonoids, stilbenes, and lignans. Figure 1 illustrates different groups of polyphenols and their basic chemical structures. 

Phenolic acid accounts for about one-third of the polyphenolic compounds in our diet and is abundant in acidic-tasting fruits. The term “phenolic acids” describes the phenolic compounds that have one carboxylic group [18]. They are mainly divided into two sub-groups, which are hydroxybenzoic and hydroxycinnamic acid [19]. The most commonly found hydroxybenzoic acids are *p*-hydroxybenzoic, protocatechuic, vanilic, and syringic acids. Hydroxybenzoic acids possess a common structure of C_6_-C_1_ and are derived from benzoic acid. On the other hand, the four most common hydroxycinnamic acids are ferulic, caffeic, *p*-coumaric, and sinapic acids [20]. Hydrobenzoic acids can be found in tea while hydroxycinnamic acids can be found in cinnamon, coffee, blueberries, kiwis, plums, apples, and cherries. These acids are mostly found as glycosylated derivatives of esters of quinic acid, tartaric acid, and shikimic acid [7].

Flavonoids are the most abundant polyphenols found in the human diet. The basic structure of flavonoids consists of two aromatic rings that are bound together by three carbon atoms forming an oxygenated heterocycle. The literature has revealed that flavonoids possess both antioxidant and anti-inflammatory properties, which are mainly found in fruits, vegetables, legumes, red wine, and green tea. Flavonoids are subdivided into six classes: flavonols, flavones, isoflavones, flavanones, anthocyanidins, and flavanols [7]. Flavonols being the most ubiquitous flavonoids in foods, are present at relatively low concentrations. Quercetin and kaempferol are the main representatives of flavonols, and are abundant in onions, curly kale, leeks, broccoli, and blueberries. Flavones, which are less common than flavonols, mainly consist of glycosides of luteolin and apigenin. Parsley and celery are the main edible sources of flavones [7]. Meanwhile, isoflavonoids that have structural similarities to estrogens possess the ability to bind to estrogen receptors and thus, are classified as phytoestrogens. Catechins and proanthocyanidins are the monomer and polymer forms of flavanols, respectively. Green tea contains a substantial amount of catechins, including gallocatechin, epigallocatechin, and epigallocatechin gallate (EGCG) [21].

Stilbenes contain two phenyl moieties that are connected by a two-carbon methylene bridge. Since stilbenes are found in low quantities in our diet, larger quantities may be needed to exert significant health effects, which can be provided in concentrated extracts or in the form of purified compounds. Resveratrol, which can be found in red wine and peanuts, is the most extensively studied stilbene. It has been extensively studied for its anticarcinogenic effect [22]. However, the protective effect of resveratrol is unlikely at normal nutritional intakes since this molecule is found in such small quantities in the diet [23].

Lignans are diphenolic compounds containing a 2,3-dibenzylbutane structure that is formed by the dimerization of two cinnamic acid residues [5]. Lignans can be found in flax seeds, legumes, cereals, grains, fruits, algae, and certain vegetables. Flax seeds are high in lignans, at about a thousand times higher than in other food sources.

It has been widely acknowledged that dietary polyphenols play important roles in human health. High intake of fruits, vegetables, and cereals, which are rich in polyphenolic molecules, has been associated with lower risks of chronic disorders [24]. Natural polyphenols have been shown to have potent antioxidant activity from their role in the inhibition of free radicals by deactivating their active species and/or precursors.

### 2.1. Antioxidant Mechanism of Action 

The main mechanism proposed for the antioxidation action of phenols includes, molecular complexation with pro-oxidant protein, chelation of potentially pro-oxidant metal ions (Fe^3+^, Al^3+^, Cu^2+^), or by direct trapping of reactive oxygen species (ROS) [25]. As a primary antioxidant, polyphenols work to inactivate free radicals through the hydrogen atom transfer (HAT), single electron transfer (SET), and transition metals chelation (TCM) [25]. 

In the HAT mechanism (1), the phenolic antioxidant, as indicated by ArOH, may react with the free radical, R, by transferring its hydrogen atom through the hemolytic rupture of the O-H bond. This reaction produces harmless RH species and oxidized ArO^−^ radicals, which are less reactive than the original reactive radicals. The main determining factor for the antioxidant action of the phenolic compound in the HAT mechanism is bond dissociation enthalpy (BDE). The lower the BDE value of the phenolic O-H bond, the easier the dissociation of the O-H bond to react with the free radicals. The dissociation energy, BDE, is determined by the presence, number, and positions of additional phenolic hydroxyl groups, their involvement in the formation of intramolecular hydrogen bonds, and the possibility of allowing electronic delocalization based on the conformation throughout the molecule [26].

In the SET mechanism (2), an electron from ArOH is donated to the free radical, R, which produces an energetically stable anion species, R^-^, and a less reactive cation radical, ArOH^+^•. The ionization potential (IP) is the important determinant for the scavenging activity evaluation in this mechanism. The lower the IP, the easier the electron abstraction [25]. The antioxidant potential efficacy of each polyphenol mechanism is determined by the two basic physicochemical parameters, BDE and IP, respectively. The stable phenoxy radicals, ArO• and ArOH^+^•, are produced by the reactions as the result of delocalization of their unpaired electron over the aromatic ring either by resonance or hyperconjugation effects [26].

Hydrogen atom transfer (HAT):

(1) $$\mathrm{ArOH}+R•\underset{\left(\mathrm{OH}\;\mathrm{hemolytic}\;\mathrm{rupture}\right)}{\to }\mathrm{ArO}•+\mathrm{RH}$$

Single electron transfer (SET):

(2) $$\mathrm{ArOH}+R•\underset{\left(\mathrm{electronic}\;\mathrm{abstraction}\right)}{\to }{\mathrm{ArOH}}^{+}•+R^{-}$$

Another antioxidative mechanism of polyphenols is via chelation of transition metals (transition metal chelation, TCM) that leads to stable complex compounds [27,28]. The antioxidant capacity of polyphenols prevents the redox-active transition metals from catalyzing free radical formation. For instance, metal ions (mainly Fe^2+^) that react with hydrogen peroxide may be inactivated by polyphenols through suppression of the superoxide-driven Fenton reaction, the harmful source of ROS [29]. Hydroxyl radical (OH•) is the most reactive oxygen radical that cannot be eliminated by enzymatic reactions and may react with any kind of substrate they encounter [30]. Hence, if these metal ions are not bound to protein or any chelators, a Fenton-like reaction may take place, causing the accumulation of free radicals and the initiation of biomolecules’ damage processes. 

The formation of relatively stable phenoxyl radicals by an antioxidant mechanism may disrupt the chain oxidation reactions in cellular compartments and protect cell constituents against oxidative damage [31]. This may limit the risks of various degenerative diseases associated with oxidative stress [32]. There are increasing evidences from animal studies that dietary supplementation with polyphenols may limit the development of cancers, cardiovascular diseases, neurodegenerative diseases, diabetes, and osteoporosis. These diseases are mainly associated with the occurrence of oxidative damage to cell components, DNA, proteins, and lipids due to aging, which contributes to the degeneration of somatic cells and the pathogenesis of the diseases.

Studies have also shown that polyphenols may induce phase II enzymes, such as glutathione *S*-transferase via nuclear factor erythoid-2-related factor 2 (Nrf2)-mediated antioxidant responsive element (ARE) pathway [33]. Polyphenols may be responsible for the release of Nrf2 from its cytosolic inhibitor, Keap1, and an increase in Nrf2 stability. The stabilization of Nrf2 is important to maintain the cellular defense mechanism [34]. This molecular basis demonstrated the role of polyphenols in protection against the development of various diseases by regulating the antioxidant/detoxifying enzymes via Nrf2 signaling. 

Apart from its antioxidant activity, a large body of evidence exists for the anti-inflammatory effects of polyphenols. The anti-inflammatory capacity of polyphenols is contributed by the following functions: firstly, by acting as antioxidants, secondly by interfering with oxidative stress signaling, and thirdly by suppressing the pro-inflammatory signaling transduction [35]. The potential molecular mechanism of their anti-inflammatory activities may also include the inhibition of enzymes related to inflammation, such as cyclooxygenase and lipoxygenase, and many others such as peroxisome proliferator-activated receptors (PPAR), nitric oxide synthase (NOS), nuclear transcription factor κB (NF-κB), and NSAID activated gene-1 (NAG-1) [36]. The modulatory effect of polyphenols on cellular biomarkers related to oxidative stress and inflammation establish the role of polyphenols in reducing the risk of many chronic diseases.

### 2.2. Health-Beneficial Effects of Polyphenols and Their Limitations

Prolonged oxidative stress could lead to chronic inflammation and chronic diseases [37]. The abundant literature has shown that polyphenols provide protection against the development of oxidative stress-related diseases. Even though cancer is one of the major causes of death in the world, it is preventable and highly susceptible to modulation by dietary factors [38]. Phenolic compounds, which are abundant in vegetables and fruits from our diet, are important chemopreventive agents. For instance, studies have demonstrated that functional food in the Mediterranean diet displayed an important role in cancer prevention by inactivating carcinogens, decreasing cell proliferation, inducing cell cycle arrest and apoptosis, and inhibiting angiogenesis in many types of tumor [39].

Many polyphenolic compounds, such as quercetin, catechin, isoflavones, lignans, flavanones, ellagic acid, curcumin, or resveratrol have been extensively studied. All of them showed protective effects in different cancer models. A substantial number of in vivo and in vitro studies have documented the cancer-preventive effect of quercetin by induction of apoptosis during different cycle stages without affecting normal cells [40]. A study by Mu et al. [41] demonstrated the therapeutic effect of quercetin on human hepatoma cell lines (HepG2), while Jeong et al. [42] evaluated the effect of quercetin in human breast carcinoma cells. These studies showed that quercetin protected cells from oxidative stress, inflammation, and DNA damage via its antioxidant properties. In addition, quercetin modulated the growth of many cancer cell lines by blocking cell cycle progression and tumor cell proliferation and by inducing apoptosis. Resveratrol is another polyphenol, which has shown preventive effects against many types of cancers, including activity on prostate, breast, and stomach cancer cell lines. It affected carcinogenesis by modulating the signal transduction pathway that controls cell division and growth, apoptosis, inflammation, angiogenesis, and metastasis [43]. However, the use of polyphenol in humans as a chemopreventive agent was limited by their poor bioavailability. For instance, resveratrol found in berries and grapes is well tolerated by humans but is rapidly metabolized, leading to a short half-life and limited effectiveness [44,45]. 

Neurodegenerative diseases, such as Alzheimer’s disease, Parkinson’s disease, and other types of dementia are dependent of oxidative stress that particularly affects brain tissues [46]. Therefore, antioxidant compounds may also contribute to the prevention of such diseases. Dietary supplementation of fruits’ or vegetables’ extracts rich in polyphenols such as blueberry, spinach, and strawberry has shown to retard and reverse age-related neuronal signal-transduction and cognitive behavioral deficits in aging rats [47,48]. Apart from that, supplementation of grape polyphenols was also shown to protect the experimental animals from the neurodegenerative changes induced by chronic ethanol consumption by preventing the decrease in synaptic protein function [49]. On the other hand, an in vitro experiment demonstrated that a low dose of green tea polyphenols is more effective in preventing neurodegenerative disease compared to higher doses, which appeared to be pro-oxidant and toxic [50]. The findings of the study have suggested that neuroprotective mechanism of green tea polyphenols against oxidative stress-induced cell death include stimulation of protein kinase C (PKC) and modulation of cell survival/cell cycle genes. However, little is known about polyphenol concentration in the brain. There is an issue on the permeability of the blood–brain barrier to polyphenols and several studies were carried out to determine polyphenols’ concentrations such as flavanone (naringin and quercetin) and isoflavone (genistein) in the plasma and tissues of rats [51,52,53]. Data from these studies have demonstrated poor blood–brain barrier penetration of naringin, genistein, or quercetin.

Osteoporosis and its fracture complication is another age-related disease that contributes to a high mortality rate in humans [54]. Apart from hormonal imbalance and chronic inflammation, osteoporosis is also associated with oxidative stress [55]. Lifestyle and dietary modification have been proposed as the natural approach to reduce the risk of osteoporosis. Recently, many studies have found the link between dietary polyphenols intake and bone health [56]. Bioactive phenolic compounds such as soy isoflavones have attracted much attention as a possible alternative agent to prevent osteoporosis. Supplementation of soybean isoflavones genistein, daidzein, or their glycosides to ovariectomised rats for several weeks prevent the loss of bone mineral density and trabecular volume [57,58]. A meta-analysis of several randomized clinical trials has revealed that supplementation of soy isoflavones could prevent postmenopausal osteoporosis and improve bone strength by increasing lumbar spine bone mineral density (BMD) and decreasing bone resorption marker (urine deoxypiridinoline), thereby decreasing the risk of fracture [59,60]. However, soy isoflavones’ supplementation did not have a significant favorable effect on BMD of total hip, femoral neck, and trochanter, as well as bone formation markers (bone alkaline phosphatase and osteocalcin) in menopausal women. Further studies are required to address factors affecting the magnitude of beneficial effects of soy isoflavones on bone [61].

Due to their potent antioxidant properties, abundance in diet, and preventive role of oxidative stress-related diseases, researchers and food manufacturers have become more interested in polyphenols [7]. These valuable properties are largely dependent on the stability, bioactivity, and bioavailability of the active ingredients [62]. Polyphenols were reported to be unstable to pH, enzymes, or the presence of other nutrients in the gastrointestinal system, thus limiting their activity and potential [26]. 

Food is conveniently taken orally but food processing into digestible form occurs in a strict sequence. The lining of the digestive tract might not be proper for the absorption of bioactive components from the food. After being swallowed and transferred into the stomach, digestion of the food components takes place in the acid solution of the stomach [63]. In fact, only a small proportion is absorbed due to short gastric residence time, low permeability, and low solubility. Therefore, these compounds require a protective mechanism that can maintain the chemical integrity of the bioactive components in order to deliver them to the physiological target [64]. 

In addition, polyphenols are sensitive to physical and chemical conditions such as light, heat, and oxidation during food processing, distribution, or storage [26]. They may oxidize very quickly, leading to the progressive appearance of brown color and/or unwanted odors with consequent loss of activities. The unpleasant taste of most phenolic compounds, which is very astringent and bitter, also limits their use in food or oral medication. Hence, it is important for product formulators and manufacturers to provide protective mechanisms that can maintain the active molecular form up to the time of consumption [64]. 

The utilization of encapsulated polyphenols or the formulation of a finished protected product instead of free compounds could effectively overcome these limitations [26,62]. This could be achieved by encapsulation technologies, such as spray drying, liposome entrapment, nanoencapsulation, freeze-drying, inclusion complexation, and cocrystallization. Through these encapsulation systems, the stability and physicochemical properties of nutrients were enhanced compared to the non-encapsulated ingredients [65]. 

## 3. Nanoencapsulation of Phenolics

Encapsulation can be defined as a process to entrap an active agent or a substance within another substance or carrier material [66]. In the food industry, encapsulation is a useful tool to improve the delivery of biomolecules (antioxidants, minerals, vitamins, lycopene, fatty acids) and living cells (antibiotic) into food. Since most of the nutraceuticals are sensitive and labile, the goal of encapsulating nutraceuticals is to reduce the damage and protect the bioactive agents from undesirable circumstances [65]. Beside bioactive protection, their bioavaibility was also improved due to an increase in surface-to-volume ratio by reducing the particle size into the nano-range [67,68].

Nanoparticles are sub-micron solid particles that may or may not be biodegradable and can be used for encapsulation of bioactive compounds [69]. Therefore, nanoencapsulation can be defined as a process of coating a substance within another material at sizes on the nano-scale, ranging from 1 to 1000 nm [69,70]. Nanocarriers increase nutraceuticals’ bioavailability by allowing them to easily enter the bloodstream from the gut [71].

Nanoencapsulation technology is a promising and a novel method that could preserve the core material from adverse environmental conditions and undesirable effects that cause their degradation through along the digestive tract [72]. Polyphenols’ nanoencapsulation could also alleviate the unpleasant tastes or flavors and overcome the drawbacks related to its instability, as well as improve the bioavailability and half-life of the compound in vivo and in vitro [62].

The selection of suitable materials and techniques for encapsulation are important for successful encapsulation of bioactive compounds. Biodegradable polymeric nanoparticles are highly preferred by many researchers from food and pharmaceutical fields due to their good properties in terms of biocompatibility, design and preparation, structure variation, and bio-mimetic characters [73]. Apart from that, they are stable in blood, have low toxicity, are non-thrombogenic, non-immunogenic, non-inflammatory, and are applicable to various molecules [74]. 

Solvent evaporation method is the main dispersion method for the preparation of polymeric nanoparticles [73]. This method can be used for nanoencapsulation of phenolic compounds such as quercetin in polymeric nanoparticles. For instance, quercetin can be loaded in polymeric nanoparticles by the solvent evaporation method to improve its poor aqueous solubility and stability. The antioxidant activity assay has revealed that the functional activity of quercetin was retained after nanoencapsulation [75]. Emulsification or solvent diffusion is another dispersion method, which is commonly used for creating nanoparticles. It has been used for loading of curcumin in PLGA nanoparticles to improve oral bioavailability of curcumin by at least 9-fold [76]. The usage of highly bioavailable nanoparticle formulation of polyphenols is expected to bring about the improvement of phenolic compounds’ efficacy as a therapeutic agent for the treatment of numerous disorders.

### 3.1. Poly(lactic-co-glycolic acid) (PLGA)-Based Nanoparticles

PLGA has been extensively studied for polyphenols’ delivery due to their biocompatible and biodegradable features. PLGA-nanoparticles are internalized in cells partly through fluid phase pinocytosis and in part through clathrin-mediated endocytosis in vascular smooth muscle cells before rapidly entering the cytoplasm [77]. Following internalization, biodegradable PLGA nanoparticles undergo surface charge reversal in the acidic pH of endo-lysosomes, which facilitates an interaction of nanoparticles with the vesicular membranes. This leads to transient and localized destabilization of the membrane, thereby resulting in the escape of nanoparticles into cytosol [78]. A significant fraction of nanoparticles underwent exocytosis and only 15% escaped into the cytosolic compartment. The fraction of nanoparticles that escape the endosomal compartment may remain in the cytoplasmic compartment and release the encapsulated therapeutic agent in a sustained manner as the polymer slowly degrades [77].

In line with the technological advancement in drug delivery systems, PLGA has been widely and successfully used to encapsulate extract of natural products, including polyphenols. A number of in vitro and in vivo studies have been performed to confirm that nanoencapsulation of polyphenolic compounds with biopolymer are useful in enhancing their protective potential. 

### 3.2. Therapeutic Potentials of PLGA-Encapsulated Polyphenols

#### 3.2.1. Anti-Inflammatory Potential

Most chronic illnesses, including cancer, diabetes, and cardiovascular diseases, are mediated through chronic inflammation [79]. Hence, the suppression of chronic inflammation may have the capacity to delay, prevent, and even treat various chronic diseases. A large number of studies have shown that dietary polyphenols were associated with anti-inflammatory activities [35]. In fact, the free radical scavenging activity of polyphenols was mainly contributed by the anti-inflammatory actions of these molecules. In recent years, encapsulation of polyphenolic compounds with PLGA nanoparticles have been extensively studied to overcome the setbacks related to their bioavailability and efficacy. 

Polyphenolic compounds, particularly catechin, found in cherry extract possessed both antioxidant and anti-inflammatory properties; however, they have low oral bioavailability. A recent study has evaluated the efficacy of polyphenol-rich cherry extracts from *Prunus avium L* encapsulated in PLGA nanoparticles [80]. Different concentrations of cherry extract were tested for its antioxidant gastrointestinal permeability using a triple-cell-co-culture model (Caco-2/HT29-MTX/RajiB), which resembles the intestine. Results from the study showed that PLGA nanoparticles were able to promote permeability of the encapsulated cherry extract while maintaining their antioxidant activity. Due to its low cytotoxicity, the use of PLGA nanoparticles could allow administration of higher cherry extract doses. Cherry extract entrapped in PLGA nanoparticles has been found to protect human umbilical vein endothelial cells (HUVECs) from oxidative stress induced by H_2_O_2_.

In another study, resveratrol-loaded galactosylated PLGA nanoparticles was evaluated for their oral bioavailability and in vitro anti-inflammatory activity, in Sprague-dawley rats and lipopolysaccharides-induced murine macrophage cell line, RAW 264.7, respectively [81]. Galactosylated PLGA nanoparticles have significantly enhanced oral bioavailability of resveratrol. In situ single-pass intestinal perfusion and cellular uptake evaluation showed that galactosylated nanoparticles could improve the intestinal permeability and transcellular transport of resveratrol. The authors indicated that resveratrol-loaded galactosylated PLGA nanoparticles could effectively promote the intestinal absorption of resveratrol and enhance its anti-inflammatory bioactivity, which may be a promising approach for the treatment of inflammatory diseases.

Wan et al. [82] investigated the effect of resveratrol-loaded PLGA nanoparticles on non-alcoholic fatty liver disease (NAFLD) therapy in HepG2 cells. NAFLD is characterized biochemically by the inactivation of 5’ adenosine monophosphate-activated protein kinase (AMPK), hepatic lipid accumulation, decreased insulin sensitivity, and inflammation [83]. Resveratrol-loaded PLGA that was prepared according to an oil/water emulsion technique exhibited better efficiency in alleviating lipogenesis, promoting lipolysis, and reducing hepatocellular proliferation than free resveratrol. The superior property of resveratrol-loaded PLGA was due to its improved stability, water solubility, and bioactivity.

As reported by Chakraborty et al. [84], PLGA encapsulated quercetin prepared using emulsion-diffusion-evaporation methods, has significantly higher potency in downregulating matrix metalloproteinase-9 (MMP-9), infiltration of inflammatory cells, and oxidative damage in rat gastric tissues, compared to free quercetin. The nanoencapsulated quercetin could also prevent higher inducible-NOS (iNOS) expression and NFκβ activation, which could lead to inflammation and cell damage in ethanol-induced gastric ulcer. 

Curcumin is an active polyphenol component isolated from turmeric roots, which possesses anti-inflammatory and antioxidant properties. The literature has shown that curcumin could behave as a universal anti-inflammatory drug but has a major drawback of poor in vivo bioavailability, due to its hydrophobic nature. A study by Betbeder et al. [85] showed that PLGA nanoencapsulated curcumin has greater antioxidant and anti-nitrosant activities in epithelial cells and in an acellular model when compared to their free form. The authors suggested that PLGA nanoparticles may create a nano-environment that concentrates and facilitates interactions of the curcumin with reactive oxygen species (ROS) and reactive nitrogen species (RNS), and hence, augmented the antioxidant and anti-nitrosant activities of curcumin.

Previous studies have shown that EGCG, which could be found in green tea, possessed very strong antioxidant and anti-inflammatory properties. A study by Srivastava et al. [86] showed that EGCG-loaded PLGA nanoparticles significantly induce DNA repair genes and inhibit the inflammatory genes. These preventive actions were deduced from 7,12-dimethylbenzanthracene (DMBA)-induced DNA damage in mouse skin using the DNA alkaline unwinding assay. The authors have demonstrated that tea polyphenol loaded with PLGA nanoparticles have a 30-fold dose advantage over the free EGCG doses in preventing DNA damage and could be used in chemoprevention.

#### 3.2.2. Anti-Cancerous Potential 

Natural products, including polyphenols, have been known for their anticancer effects for a long time. A significant number of in vitro and in vivo studies have illustrated the protective role of polyphenols against cancer due to their ability to interfere with the carcinogenesis process [5]. Apart from that, the therapeutic efficacy of polyphenols is also linked to their synergistic effect with conventional drugs for several cancer treatments [87]. In recent years, a wide range of biocompatible polymers has been utilized as polymeric nanoparticles to deliver anti-cancer drugs. Encapsulation of polyphenolic compounds with synthetic polymer PLGA nanoparticles was also investigated in light of enhancing the efficacy of their chemotherapeutic effects.

Quercetin and catechin, which are considered as important bioflavonoid polyphenols, have great potential as antioxidant, anti-mutagenic, and anti-carcinogenic agents [88]. Pool et al. [89] proved that the antioxidant effects of quercetin and catechin were enhanced by the incorporation with PLGA nanoparticles. The antioxidant capacity measured by superoxide anion-scavenging activity, lipid peroxidation assay, and chelating activity, showed that PLGA-encapsulated quercetin has more potent antioxidant action against peroxyl radical-induced lipid peroxidation and greater chelating activity towards transient metals than non-encapsulated quercetin. The results obtained from the study suggested that these delivery systems may be suitable to increase the shelf life of bioactive flavonoids and may be helpful as a therapeutic system to delay the development of oxidative stress-related diseases, including cancer.

Quercetin has also shown great potential in reducing adverse side effects and enhancing anti-tumor efficacy of chemotherapeutic drugs. PLGA-encapsulated polyphenols in combination with other anticancer drugs, such as cisplatin and tamoxifen, have been investigated for its oral bioavailability and therapeutic improvements. In their study, Jain et al. [90] investigated co-encapsulation of quercetin and tamoxifen in polymeric nanoparticles and their implications on oral bioavailability, anti-tumor efficacy, and drug-induced toxicity. The results demonstrated that encapsulation with PLGA nanoparticles prepared by the emulsion-diffusion evaporation method possessed better oral delivery, ~3-fold, which was about a 3-fold increase in oral bioavailability as compared to the free form quercetin. The authors also showed that in contrast to free drugs’ combination, co-encapsulation with nanoparticles resulted in higher anti-tumor efficacy, as revealed by higher tumor suppression when tested against a DMBA-induced breast cancer model in female Sprague-Dawley rats. Co-encapsulation of quercetin and tamoxifen with PLGA nanoparticles could be a promising approach in improving oral delivery of quercetin and tamoxifen for cancer therapy.

The anticancer potential of polymer-based nanoparticles of EGCG and theaflavin alone or in combination with cisplatin have been studied by Singh et al. [91] in human cancer lines, A549 (lung carcinoma), HeLa (cervical carcinoma), and THP-1 (acute monocytic leukemia), using the cell proliferation assay and cell cycle assay. After nanoencapsulation with PLGA, polyphenols alone or in combination with an anticancer drug, were found to be more effective in inhibiting cell proliferation, metastasis, angiogenesis, and apoptosis biomarkers. The results showed that the nanoencapsulated polyphenols exhibit ~20-folds, about a 20-fold dose advantage in exerting their anticancer effect compared to native EGCG and theaflavin in A549, HeLa, and THP-1 cells. The researchers demonstrated that PLGA-mediated delivery of polyphenols could serve as a basis for enhancing bioavailability and limiting the unwanted toxicity of chemotherapeutic agents. 

Nassir et al. [92] have studied the effect of encapsulated resveratrol in PLGA nanoparticles on their cytotoxic and mode of apoptotic cell models’ death against prostate cancer cell lines. The results showed that encapsulation of resveratrol with PLGA nanopolymer induced apoptosis in prostate cancer cell lines with no adverse effect on normal macrophage cells. This was through the evaluation of the MTT cytotoxic assay and combined use of different apoptotic markers, such as phosphatidylserine externalization, the TUNEL assay, analysis of mitochondrial membrane potential, cell cycle status, the caspase-3 assay, and assessment of ROS generation. In addition, resveratrol nanoparticles exhibited significantly greater cytotoxicity towards prostate cancer cell lines compared to free resveratrol. No adverse cytotoxic effects on murine macrophages were observed even at the highest dose. The authors supported the potential developing and use of resveratrol-loaded nanoparticles for prostate cancer chemoprevention.

#### 3.2.3. Neuroprotective Potential

Polymeric materials, including PLGA, were among the first to be used to transport drugs to the brain [93]. Nanoparticles for brain application have dimensions of fewer than 200 nm. The small aggregation size conferred a high degree of tissue penetration. Nanotechnology may be able to tackle the limited efficacy of polyphenols applications for the treatment and management of many disorders, including brain diseases. Other than improving the aqueous solubility and bioavailability of drugs, a nanoparticle-based delivery system also may overcome physiological barriers [94,95]. Polyphenols have low bioavailability in the brain due to their limited capacity to cross the blood–brain barrier. Hence, the use of polymeric-based nanotechnology to deliver natural polyphenolic compounds across the blood–brain barrier seems to be a promising strategy for neurodegenerative diseases’ prevention and treatment.

Studies have demonstrated that curcumin-loaded nanoparticles were an effective and attractive treatment for brain diseases, particularly Alzheimer’s disease, Parkinson’s disease, and cancer. Szymusiak et al. [96] proved that the incorporation of highly hydrophobic curcumin into stable polymeric nanoparticles, PLGA could enhance its oral absorption. Pharmacokinetic analysis after oral delivery of nano-curcumin demonstrated that the dose requirement was reduced approximately 2-fold to achieve comparable plasma and central nervous system tissue concentrations in mice when compared to non-encapsulated curcumin.

More recently, the effect of PLGA nanoparticles loaded with curcumin on neuronal differentiation and neuronal stem cells’ proliferation were explored in a study by Tiwari et al. [97]. Curcumin-encapsulated PLGA nanoparticles were shown to potently induce neuronal stem cells’ proliferation and neuronal differentiation in vitro and in adult rats’ hippopocampus and subventricular zone, as compared to uncoated curcumin. Based on transmission electron microscopy analysis, the PLGA-nanoencapsulated form of curcumin induced neurogenesis by internalization into the hippocampus. Curcumin nanoparticles significantly increased expression of the genes involved in cell proliferation and neuronal differentiation. The results also demonstrated that PLGA-encapsulated curcumin reversed the deficits in hippocampal neurogenesis and in learning and memory dysfunction in an Alzheimer’s disease rat model. Curcumin nanoparticles induced neurogenesis by activation of the canonical Wnt/β-catenin pathway and enhanced the brain self-repair mechanism. PLGA-encapsulated curcumin could be a promising therapeutic approach in treating neurodegenerative diseases such as Alzheimer’s disease.

As is widely described in previous studies, quercetin may exert a protective action against Alzheimer’s disease and other oxidative stress-related neurodegenerative diseases [98,99]. However, Ansari et al. [99] showed that quercetin acted as an antioxidant at lower doses, but was neurotoxic at higher doses. The neuroprotective effect of quercetin was also limited by its poor blood–brain barrier permeability. Ghosh et al. [100] evaluated the therapeutic efficacy of PLGA-nanoencapsulated quercetin in combating ischemia-reperfusion-induced neuronal damage in young and aged Swiss Albino rats. It was demonstrated that three days of continuous reperfusion after ischemia caused massive damage to neuronal cells. Upregulation of inducible nitric oxide synthase (iNOS) expression may lead to excessive nitric oxide (NO) production, which in turn may react with superoxide to form peroxynitrite, a powerful radical that induced neuronal death after cerebral ischemia [101]. Oral treatment of nanoencapsulated quercetin was found to downregulate iNOS and caspase 3 activities and improve neuronal count in the hippocampal subfields. The results suggested that PLGA nanoencapsulation could be a potential approach to deliver quercetin to the brain and provide the protection against oxidative stress causing ischemic neuronal damage.

To date, there is no published clinical trial on nanotechnology-based polyphenol delivery systems in neurodegenerative diseases. Preclinical in vivo and in vitro studies suggested that a polymeric nanoparticle-based polyphenol delivery system could enhance the absorption of phenolic compounds and improve their ability to cross the blood–brain barrier. PLGA nanoparticles could also enhance the characteristic of polyphenols to overcome limitations with conventional administration. This could be a good strategy for the prevention and treatment of neurodegenerative diseases.

#### 3.2.4. Anti-Osteoporosis Potential

The anti-osteoporosis properties of polyphenolic compounds may be augmented when combined with a special delivery system, such as PLGA nanoparticles. Ahn et al. [102] was the first to demonstrate the effect of curcumin-loaded PLGA nanoparticles against bone loss in ovariectomised rats. There were improvements in bone mineral density and trabecular microarchitecture in rats fed with curcumin-loaded nanoparticles compared to non-encapsulated curcumin. Quantitative real-time PCR analyses of osteogenesis/osteoclast-related genes in the tibia of ovariectomised rats revealed that curcumin nanoparticles significantly improved bone remodeling. The authors suggested that encapsulation with PLGA nanoparticles could enhance the protective effect of curcumin against ovariectomised-induced bone loss. 

In another study by Khalil et al. [103], PLGA-encapsulated *Citrus medica* leaves extract that is rich in flavonoids was combined with rosiglitazone to prevent osteoporosis induced by diabetes in rats. Combined therapy of rosiglitazone and *Citrus medica* nanoparticles reduced the risk of osteoporosis in insulin-resistant rats. The osteoprotegerin (OPG), receptor activator of nuclear factor kappa-B ligand (RANKL), and β2-microglobulin levels were much improved in diabetic rats receiving *Citrus medica* nanoparticles. The BMD data also showed that citrus flavonoids prevented both cortical and trabecular bone loss of the femur.

Studies on the effects of encapsulating polyphenolic compounds with PLGA nanoparticles against osteoporotic bone loss are still lacking. The available studies have demonstrated that polymeric nano-technological techniques may increase the delivery and efficiency of polyphenolic compounds as anti-osteoporosis agents. This could open the door for future research in improving the therapeutic index of polyphenolic compounds against osteoporosis. Conjugation of polyphenolic compounds with PLGA nanoparticles is expected to exert higher efficacy for treatment of bone loss compared to their native form.

## 4. Conclusion and Perspectives

Despite the growing evidence regarding the beneficial effects of dietary polyphenols in the prevention and treatment of various chronic diseases, their low bioavailability represents a critical limitation for clinical application. The insufficient number of clinical trials on polyphenols as treatment of oxidative stress-related diseases, and failure to reproduce the therapeutic effects seen in in vitro models, pose major challenges. Medical and pharmacology fields have made some progress to provide innovative nanotechnology-based systems for the improvement of drug delivery and cell targeting. There is emerging evidence that nanoparticle-based delivery systems could provide solutions to polyphenol solubility, instability, and poor bioavailability. 

The sustained release of the therapeutic agent from PLGA-based nanoparticles could increase treatment efficacies. PLGA nanoparticle-based polyphenol delivery systems have been developed with biocompatibility and biodegradability characteristics. Nevertheless, their toxicity and safety should be determined first before introduction into clinical practice. The identification of possible long-term side effects is particularly important when substantial amounts of nanoparticles will be dispensed to cells or animals. The high cost of production and difficulty of scale-up also limit their use in clinical trials.

The positive results obtained thus far suggest that a nanotechnology-based system is a promising strategy to maximize the therapeutic potential of polyphenols against various diseases, including cancers, neurodegenerative, and metabolic disorders. More advanced research on polymeric nano-technology-based polyphenol delivery systems is warranted before they could be used to treat oxidative stress-related diseases.
